# MICOP: Maximal information coefficient-based oscillation prediction to detect biological rhythms in proteomics data

**DOI:** 10.1186/s12859-018-2257-4

**Published:** 2018-06-28

**Authors:** Hitoshi Iuchi, Masahiro Sugimoto, Masaru Tomita

**Affiliations:** 1 Systems Biology Program, Graduate School of Media and Governance, Keio University, Fujisawa, 252-8520 Japan; 2 Institute for Advanced Biosciences, Keio University, Tsuruoka, 997-0052 Japan; 3 Health Promotion and Preemptive Medicine, Research and Development Center for Minimally Invasive Therapies, Tokyo Medical University, Shinjuku, Tokyo, 160-0022 Japan; 4 Department of Environment and Information Studies, Keio University, Fujisawa, 252-8520 Japan

**Keywords:** Circadian rhythm, Mutual information, Proteomics

## Abstract

**Background:**

Circadian rhythms comprise oscillating molecular interactions, the disruption of the homeostasis of which would cause various disorders. To understand this phenomenon systematically, an accurate technique to identify oscillating molecules among *omics* datasets must be developed; however, this is still impeded by many difficulties, such as experimental noise and attenuated amplitude.

**Results:**

To address these issues, we developed a new algorithm named Maximal Information Coefficient-based Oscillation Prediction (MICOP), a sine curve-matching method. The performance of MICOP in labeling oscillation or non-oscillation was compared with four reported methods using Mathews correlation coefficient (MCC) values. The numerical experiments were performed with time-series data with (1) mimicking of molecular oscillation decay, (2) high noise and low sampling frequency and (3) one-cycle data. The first experiment revealed that MICOP could accurately identify the rhythmicity of decaying molecular oscillation (MCC > 0.7). The second experiment revealed that MICOP was robust against high-level noise (MCC > 0.8) even upon the use of low-sampling-frequency data. The third experiment revealed that MICOP could accurately identify the rhythmicity of noisy one-cycle data (MCC > 0.8). As an application, we utilized MICOP to analyze time-series proteome data of mouse liver. MICOP identified that novel oscillating candidates numbered 14 and 30 for C57BL/6 and C57BL/6 J, respectively.

**Conclusions:**

In this paper, we presented MICOP, which is an MIC-based algorithm, for predicting periodic patterns in large-scale time-resolved protein expression profiles. The performance test using artificially generated simulation data revealed that the performance of MICOP for decaying data was superior to that of the existing widely used methods. It can reveal novel findings from time-series data and may contribute to biologically significant results. This study suggests that MICOP is an ideal approach for detecting and characterizing oscillations in time-resolved *omics* data sets.

**Electronic supplementary material:**

The online version of this article (10.1186/s12859-018-2257-4) contains supplementary material, which is available to authorized users.

## Background

The circadian rhythm, which involves oscillations over a cycle lasting 24-h, plays a critical role in biological systems [1]. Transcriptional negative feedback loops composed of clock genes are a key component of this mechanism [1–3]. These clock genes regulate downstream gene expression, leading to the 24-h cyclic oscillation of various physiological phenomena such as cell division, energy metabolism, blood pressure, and sleep [4, 5]. Many molecules are involved in these systems, so comprehensive and multilayered approaches are required to clarify the complex systems. Thus, it is crucial to obtain a deep understanding of the circadian rhythm in order to understand biological systems.

The availability of biological time-course data is key to elucidating circadian rhythms, but there are several difficulties in analyzing biological time-series data. In particular, the accumulation of time-series omics data via the technological innovation of mass spectrometry and DNA sequencers has led to the following problems: (1) low sampling frequency and (2) unstable oscillation. The first problem is derived from the generally low sampling frequency of *omics* datasets because comprehensive approaches such as proteomics and transcriptomics are often expensive and laborious. Several *omics* studies collected time-course data every 2–4 h per day and estimated periodicity using 12 to 24 points [6–9]. This sampling frequency of *omics* data was relatively low compared with those for locomotor activity or tissue luminescence, which were provided every minute [10]. The second problem is the unstable oscillation (such as amplitude decay) of time-course experimental values. There are various types of unstable oscillations in the expression pattern of genes and proteins. For example, previous reports assumed unstable oscillations such as cosine with outlier time points, cosine with a linear trend, cosine with an exponential trend, and decaying cosine as possible natural oscillation phenomena [11, 12]. These unstable oscillations hamper oscillation detection, in particular for amplitude decay, which is often observed in experimental systems and, is caused by degradation of the metabolic activity of cells and degradation of fluorescent protein [13]. Therefore, novel computational analysis that functions over the time course of *omics* studies with limited sampling points and amplitude decay should be developed.

Many analytical approaches to predict molecules with oscillating levels from time-series data have been developed. These algorithms were classified into time-domain and frequency-domain methods [14]. Typical time-domain methods are based on cosine curve-based pattern matching and their simple algorithm helps biologists to evaluate their analytical results [14]. For example, COSOPT and chi-squared periodogram are algorithms employing curve fitting and autocorrelation, respectively [15, 16]. Hughes et al. developed a nonparametric approach using rank by the nonparametric Jonckheere–Terpstra (JT) test and obtained the strength of correlation by Kendal’s tau test (JTK) [17]. However, they have disadvantages, such as sensitivity to noise and outliers, and being able to detect only cosine wave-like curves; as such, there is a need for a novel algorithm that can overcome these obstacles. Meanwhile, frequency-domain methods based on spectral analysis are strongly noise-resistant and model-independent [14]. Fisher’s G-test estimates periodicity by calculating the periodogram of experimental data and calculating the *P-*value using Fisher’s *G*-statistic [18]. Autoregressive spectral (ARS) analysis is an approach combining time-domain and frequency-domain methods, used to identify molecules with rhythmically oscillating levels in large-scale time-resolved profiles by autoregressive spectral analyses [19, 20]. Similarly, an approach combining autocorrelation and spectral analysis after removing noise from raw data with a digital filter was also proposed [21]; however, frequency-domain methods are limited by the low sampling frequency and short time period in *omics* experiments, which means that they are often insufficient to predict the periodicity of large-scale *omics* datasets [22]. Therefore, developed approaches to characterize oscillating molecules in biological data have been used with success and have contributed to our understanding of biological systems; meanwhile, it has been shown that each method sometimes produces inconsistent results because of noise, sampling rate, and waveform [23]. A novel oscillation prediction method compatible with *omics* experiments, having a low sampling frequency, was required, for which quantitative evaluation of the performance could also be achieved.

This study developed Maximal Information Coefficient (MIC)-based Oscillating Prediction (MICOP) for analyzing time-series *omics* datasets with high-level noise and possible decay. MICOP offers unsurpassed performance to identify and characterize oscillating molecules in *omics* datasets.

## Methods

### Datasets

Time-resolved data from biological samples are generally obtained every 2–6 h per day [6–9]. Therefore, we simulated time-series data containing 6–24 points for two cycles for a performance test. Half of these artificially simulated data did not feature oscillation, while the other half did. For oscillating data, to mimic experimental data, noise according to the normal distribution (average = 0, standard deviation = 0–0.6) was added to the sin curve. The decaying time-series datasets were designed so that the value of the peak in the second cycle is one-third of the value of the peak in the first cycle. The nonoscillating data were random numerical data. Proteomics datasets of C57BL/6 J and C57BL/6, which was already normalized, were downloaded from journal websites [8, 9]. The simulated data released by Wu et al. are included in MetaCycle, as described below [23, 24].

### Design

**Fig. 1:**
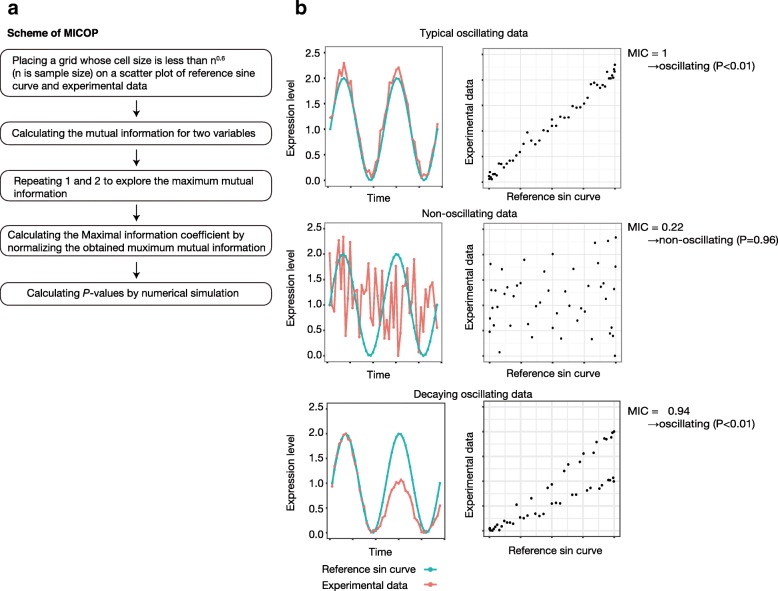
Concept of MICOP. A conceptual diagram of MICOP is shown. **a** Scheme of MICOP, **b** Typical results of MICOP. Left boxes: experimental data (red) and reference sin curves (blue); right boxes: scatter plots between reference sin curve (x-axis) and experimental data (y-axis); top: typical oscillating data (MIC = 0.1, *P* < 0.05); middle: nonoscillating data (MIC = 0.22, *P* > 0.05); bottom: decaying oscillating data (MIC = 0.94, *P* < 0.05)

A conceptual diagram of MICOP is shown in Fig. 1. The MIC belongs to the nonparametric exploration class, and the score indicates the strength of the linear or non-linear association between variables. First, the mutual information for a scatterplot of X and Y is calculated as:

$$ I\left(X;Y\right)=\sum \limits_Y\sum \limits_Xp\left(X,Y\right){\log}_2\frac{p\left(X,Y\right)}{p(X)p(Y)} $$

Where *p(X)* and *p(Y)* are marginal probability distribution functions of *X* and *Y*, and *p(X,Y)* is joint probability distribution function. Then, to compare the values from different grids and to obtain normalized values between 0 and 1, MIC is divided by the lesser number of X and Y bins. MIC is calculated as;

$$ MIC\left(X,Y\right)=\underset{X,Y<{n}^{\alpha }}{\max}\frac{I\left(X;Y\right)}{\log_2\left(\mathit{\min}\left(X,Y\right)\right)} $$

The algorithm calculates the MIC value between the reference sin curve and experimental data. The same sin curve was used for all input traces. The script for MICOP and its performance test is provided as an R script. The *P*-values were calculated from the frequency of each MIC value of experimental data and the MIC values that were calculated from the random numbers. The MIC represents the strength of association between the two variables. The MIC between the reference sin curve and targeted data, such as experimental data or simulated data, was calculated using the following steps. Step 1: Grids with different resolutions are introduced to separate the different areas of the scatter plot of the two variables. Step 2: Maximized mutual information at each resolution is selected. Step 3: The mutual information is normalized for each resolution. Step 4: The maximum value among all division methods is MIC. Step 5: to calculate the *P*-value, MIC between the reference curve and 1000 nonoscillating time-series datasets, which comprised random values, was calculated. We compared MIC values and enumerated the occurrences (*k*) when the MIC score exceeded the score calculated. *k*/1000 was taken as the *P*-value of the MICOP. Then, we compute the *P*-value as;

$$ P=\frac{1}{1000}\sum \limits_{i=1}^{1000}I\left( MIC\left( Xpi, Ypi\right)> MIC\left(X,Y\right)\right) $$

where *I* is the indicator function, and X_pi_ and Y_pi_ is the *i*th permutated version of X and Y, respectively. If the datasets have missing points, MIC is calculated without the point.

### Performance test

To test the performance of MICOP, the periodicity of simulated data was determined by MICOP, JTK, ARS, and LS. To compare the precision and sensitivity of MICOP, the MCC was compared [25]. MCC values were calculated as below:

$$ MCC=\frac{\mathrm{TP}\times \mathrm{TN}-\mathrm{FP}\times \mathrm{FN}}{\sqrt{\left( TP+ FP\right)\left( TP+ FN\right)\left( TN+ FP\right)\left( TN+ FN\right)}} $$

where TP is the number of true positives, TN is the number of true negatives, FP is the number of false positives, FN is the number of false negatives. The false discovery rate is widely used and is calculated from true positive and false positive values. In contrast, MCC is more informative as a value evaluating the performance of the classification method because it is calculated from true positive, false positive, true negative, and false negative values.

#### Reanalysis of proteomics data

To verify the practicality of MICOP, we reanalyzed the published time-series data [8, 9, 26]. Briefly, these are proteome datasets of mouse liver sampled every 3 h for 2 days, and simulated data which are two cycles containing 20 molecules [26]. The MIC and *P*-value were calculated as described in the Design section.

### Programming language, packages, and statistical analysis

R language (ver. 3.3.2) was used for all analyses [27]. Three different random seeds were used; rnorm function was used to generate random numbers according to a normal distribution and runif function was used to generate uniform random numbers. The performance of each method was compared to MICOP by Tukey-Kramer test. The *P-*values were corrected by the Benjamini–Hochberg procedure for multiple testing. A graphical package named ggplot 2 (ver. 2.2.0) was used to draw figures. The Minerva package (ver. 1.4.3) was used to calculate the MIC score, and binning range to calculate MIC score was 0.6, which is a default value of the R library. The MetaCycle package (1.1.0) was used for periodicity judgment by ARS, JTK, and LS [21, 23, 24].

## Results

### Comparison of MICOP and existing methods for decaying data

**Fig. 2:**
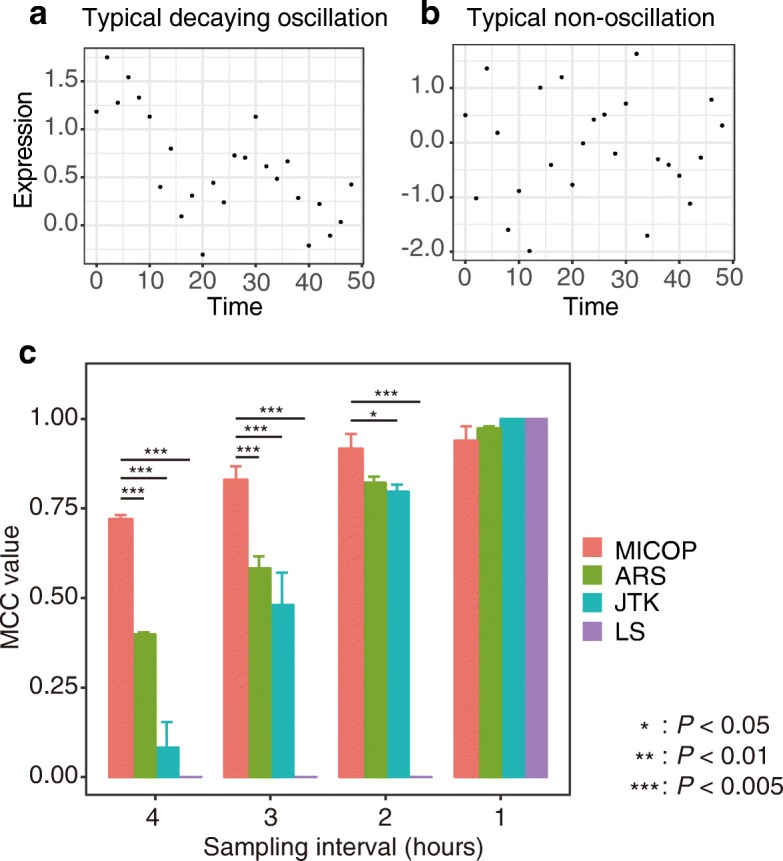
MCC values of MICOP, ARS, JTK, and LS for decaying data. Comparison of detection power of MICOP and existing methods for decaying data. **a** Typical decaying oscillation data, **b** typical non-oscillation data, **c** MCC values from simulated time-resolved data in which half represent oscillating data, whereas the other half represent random numerical data, of which half do not oscillate. Noise level was 0.4 (standard deviation). The x-axis represents the MCC value, while the y-axis represents the sampling interval (hours). The color indicates each method: red, MICOP; green, ARS; blue, JTK; and purple, LS

To test the performance of MICOP, JTK, ARSER, and Lomb-Scargle (LS) for mimicking the decaying time-resolved data, the *Matthews correlation coefficient* (MCC) values were calculated to differentiate significantly oscillating data from nonoscillating data using time-series simulation data, including 100 sets of oscillating data and 100 sets of nonoscillating ones (Fig. 2, Additional file 1) [17, 20]. Two-way ANOVA with Method and sampling frequency as factors revealed significant effects of Method (F = 631.8, *P* < 0.005), sampling frequency (F = 810.1, *P* < 0.005) and Method x sampling frequency interaction (F = 122.9, *P* < 0.005). MCC values were 0.72 (*P* < 0.005), 0.40 (*P* < 0.005), 0.082 (*P* < 0.005), and 0.00 (*P* < 0.005) for MICOP, ARS, JTK, and LS, respectively, when the sampling interval was 4 h (Fig. 2). The MCC values increased as the sampling frequency increased, and these values became almost equal to 1 in all methods at 1-h interval sampling. The MCC values of MICOP were 0.7 or more at all sampling frequencies and were the highest at a sampling interval of 1–3 h, followed by ARS and JTK. LS did not function as a classifier at a sampling interval of 1–3 h.

### Comparison of MICOP and existing methods for noisy or low-sampling-frequency or one-cycle data

**Fig. 3:**
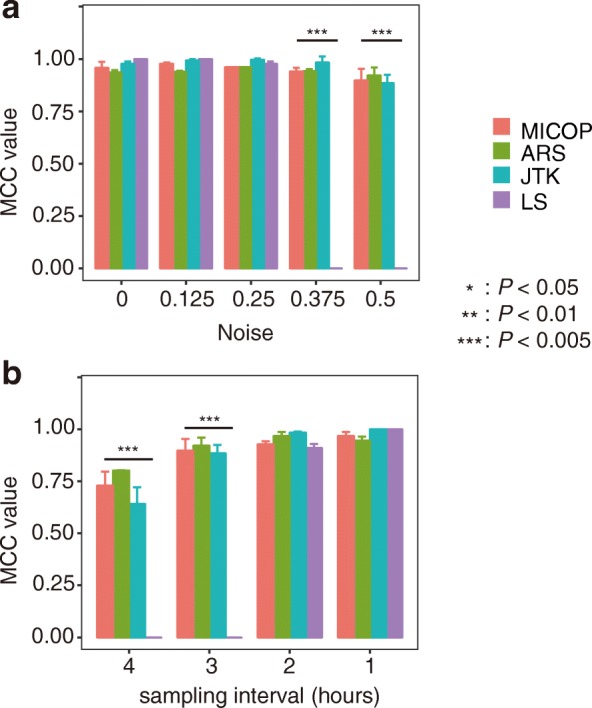
MCC values for time-series data with different sampling frequencies or gradually added noise without attenuation. Comparison of MCC values of each method when noise was added gradually (3-h sampling frequency) (**a**) and when the sampling frequency was changed (noise level was 0.4) (**b**), and both simulation data sets were not decaying. The *P-*value calculated by Tukey-Kramer test. The error bar indicates standard deviation (*n* = 3)

We compared the accuracy of MICOP and existing methods for time-series data containing noise and having a low sampling frequency without attenuation (Fig. 3a and b, Additional file 2). Initially, we quantitatively evaluated the degradation of classification performance due to the noise of MICOP (Fig. 3a). Two-way ANOVA with Method and noise level as factors revealed significant effects of Method (F = 1099.4, *P* < 0.005), noise level (F = 643.2, *P* < 0.005) and method x noise level interaction (F = 475.5, *P* < 0.005). The MCC values were 0.8 or more, except for LS, in all conditions, even if the noise was 0.500; however, LS did not function as a classifier when the noise was 0.375 or more.

The performance of MICOP as a classifier for low-sampling-frequency unattenuated data was also quantitatively evaluated (Fig. 3b). Two-way ANOVA with Method and sampling frequency as factors revealed significant effects of Method (F = 424.3, *P* < 0.005), sampling frequency (F = 447.7, *P* < 0.005) and Method x sampling frequency interaction (F = 142.2, *P* < 0.005). The MCC values increased in all methods as the sampling interval decreased, and were equal to 1 in all four methods at a sampling interval of 1 h. LS did not function as a classifier at sampling intervals of 3–4 h. The MCC values of MICOP were 0.7 or more under all conditions.

**Fig. 4:**
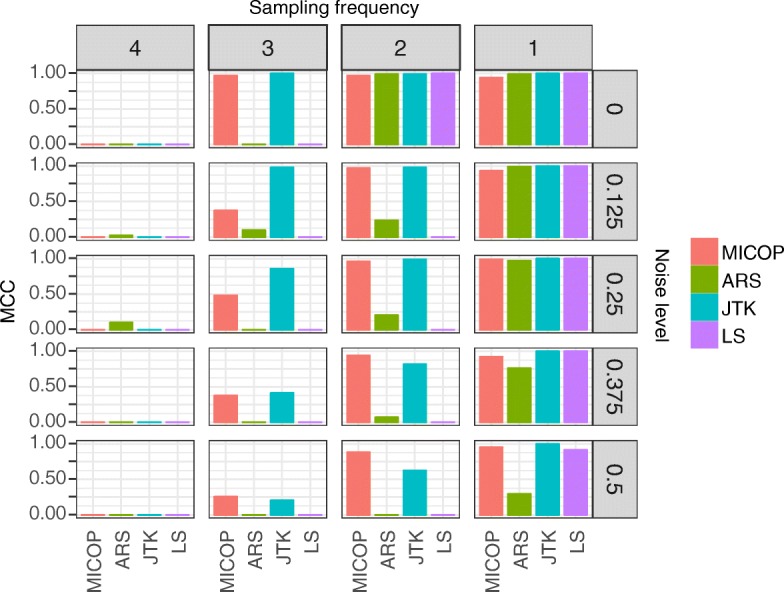
MCC values for time-series data with one-cycle data. Comparison of MCC values for each method with one-cycle time-series data. Row order indicates noise level and column order indicates sampling interval (h). Colors refer to each method. Sampling frequency and noise level were gradually adjusted

We compared the accuracy of MICOP and existing methods for one-cycle data (Fig. 4). Among all conditions (method, noise, and sampling frequency), determination accuracies using one-cycle were lower than those using two cycles. All methods did not work under all conditions at the 4-h sampling frequency. Meanwhile, MICOP and JTK showed high performances under sampling conditions ≤3 h.

### Reanalysis of previously reported time-resolved proteomics datasets

**Fig. 5:**
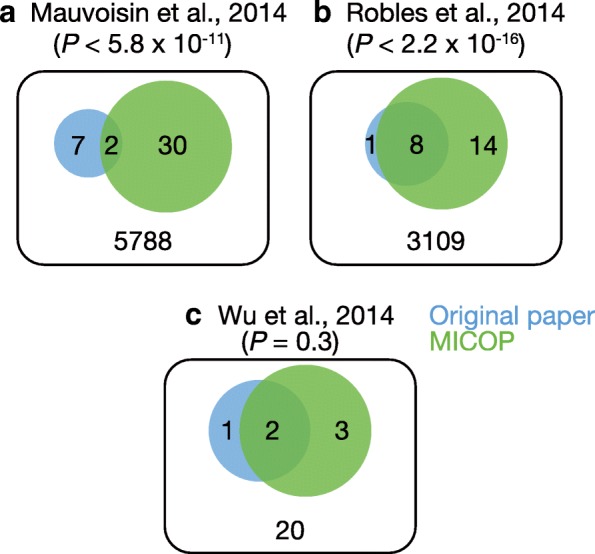
Venn diagrams of significant molecules the levels of which oscillate. Published time-resolved data sets were reanalyzed by MICOP, and Venn diagrams were constructed to quantify the overlap between MICOP and the original article. **a** and **b** represent mouse proteomics data: **a** C57BL/6 J [9], **b** C57BL/6 [8], and **c** Wu’s simulated data [23]. Blue indicates original article and green indicates MICOP. *P*-values were calculated by Chi-square tests to analyze overlap between MICOP and the original research article

We reanalyzed the time-series proteome data for mouse liver reported by Mauvoisin et al. using C57BL/6 and those reported by Robles et al. using C57BL/6 J, as well as simulated data released by Wu et al. (Fig. 5, Table 1, Table 2) [8, 9, 23]. The numbers of significantly oscillating proteins assessed by standard harmonic regression were 9 (the *F* test for multilinear regression, *P* < 0.01), 9 (Fisher’s exact test, *P* < 0.01), and 3 (*P* < 0.01) for biological data in the original work. Meanwhile, 32, 22, and 5 proteins were judged as being significantly oscillating for C57BL/6 J, C57BL/6, and Wu’s simulated data by MICOP, respectively (*P* < 0.05). The numbers of proteins judged to be significantly oscillating in both the original work and MICOP were 2, 8, and 2 for biological data, respectively. The numbers of proteins judged as being significantly oscillating for the three above-mentioned tests only by MICOP were 30, 14, and 3 for biological data, respectively.

**Table 1:** Novel oscillating protein candidates of C57BL/6 J [9] detected by MICOP

Gene name	Species	Condition	Tissue	Reference
*Acot8*	–	–	–	–
*Acox1*	*Homo sapiens*	LL	blood	[36]
	*Mus musculus*	LD	liver/SCN	[37, 38]
*Acsl5*	*Mus musculus*	DD/LD	liver/SCN	[39]
	*Mus musculus*	LD	SCN	[37]
*Akr1c14*	*Mus musculus*	LD	liver	[40]
	*Mus musculus*	DD	cartilage tissue	[41]
*Cbs*	*Mus musculus*	DD/LD	liver	[42–45]
	*Mus musculus*	LD	SCN	[37]
	*Homo sapiens*	LL	blood	[36]
*Cct8*	*Mus musculus*	LD	SCN	[37]
*Ces1b*	–	–	–	–
*Chid1*	*Mus musculus*	DD/LD	liver/SCN	[46]
*Cxadr*	*Mus musculus*	DD/LD	liver/SCN	[42]
*Cyp4f14*	–	–	–	–
*Gns*	*Homo sapiens*	LL	blood	[36]
	*Mus musculus*	DD/LD	liver	[40]
*Golgb1*	*Mus musculus*	DD/LD	liver/SCN	[37, 40, 47]
*Gpx3*	–	–	–	–
*Hars*	*Mus musculus*	LD	liver	[40]
*Hrg*	–	–	–	–
*Mfap4*	–	–	–	–
*Mug1*	*Mus musculus*	DD	liver	[42]
*Pdcd6*	*Mus musculus*	LD	liver/SCN	[37, 40, 47],
*Ptms*	*Mus musculus*	LD	SCN	[37]
*Safb*	–	–	–	–
*Serpina6*	*Mus musculus*	DD/LD	liver	[44]
*Sf3b2*	*Mus musculus*	LD	telogen epidermis	[46]
*Slc9a3r1*	*Mus musculus*	DD/LD	liver	[46]
*Snrpd3*	*Mus musculus*	LD	liver	[47]
*Stk38*	*Mus musculus*	DD	liver	[46]
	*Mus musculus*	LD	SCN	[37]
*Tpr*	*Mus musculus*	DD/LD	liver	[40, 46]
*Txndc15*	*Mus musculus*	DD/LD	liver	[46, 47]
	*Mus musculus*	LD	SCN	[37]
*Ubl4a*	*Mus musculus*	LD	SCN	[37]
	*Mus musculus*	DD	liver	[46]
*Uox*	*Mus musculus*	DD/LD	liver	[39, 40, 42, 47]
*Ythdf2*	*Mus musculus*	LD	liver	[47]

Novel oscillating protein candidates identified by MICOP from time-series proteomics data of C57BL/6 J [9] and a list of previous papers that have experimentally demonstrated that gene expression oscillates in transcriptome analysis. LD stands for the daily 24-h light-dark (LD) cycle and DD stands for constant darkness conditions. Hyphens indicate that we could not find previous consistent works which prove the mRNA oscillation

**Table 2:** Novel oscillating protein candidates of C57BL/6 [8] detected by MICOP

Gene name	Species	Condition	Tissue	Reference
*Anp32e*	*Mus musculus*	DD/LD	liver	[39, 40]
*Anpep*	–	–	–	–
*Cgn*	*Mus musculus*	LD	liver	[47]
*Csde1*	*Mus musculus*	DD	liver	[39]
	*Mus musculus*	LD	SCN	[37]
*Enpp4*	*Mus musculus*	LD	liver/anagen epidermis	[40, 46, 47]
*Gnl2*	*Mus musculus*	DD	hippocampus/liver	[39, 48]
	*Mus musculus*	LD	SCN	[37]
*Ldhb*	*Homo sapiens*	LL	blood	[36]
	*Mus musculus*	LD	anagen epidermis, SCN	[37, 46]
*Numa1*	*Mus musculus*	LD	liver	[40]
	*Mus musculus*	DD	cartilage tissue	[41]
*Prdx2*	*Mus musculus*	LD	SCN	[37]
*Rnf114*	–	–	–	–
*Slc4a1*	–	–	–	–
*Slco1b2*	*Mus musculus*	DD/LD	liver	[39, 40, 42]
*Tomm70a*	–	–	–	–
*Vps26a*	–	–	–	–

Novel oscillating protein candidates identified by MICOP from time-series proteomics data of C57BL/6 [8] and a list of previous papers which experimentally demonstrated that gene expression oscillates in transcriptome analysis. LD stands for the daily 24-h light-dark (LD) cycle and DD stands for constant darkness conditions. Hyphens indicate that we could not find previous consistent works which prove the mRNA oscillation

## Discussion

Although many algorithms have been developed to extract molecules with rhythmic oscillation in their levels from large-scale time-series data derived from mass spectrometry systems or DNA sequencers, it is known that the accuracy and sensitivity of such methods depend on noise, sampling frequency, and waveform. In particular, the discussion of the prediction power in conditions of decaying oscillation was insufficient. In this research, we provide MICOP, which is classified as a time-domain method, and demonstrate that the algorithm is particularly effective for detecting decaying oscillation.

We compared the detection power of MICOP and previously reported algorithms for decaying oscillation. We revealed that, in terms of the power for detection decaying oscillation, MICOP outperformed other algorithms (Fig. 2). In particular, MICOP showed a clear advantage when the sampling frequency was low. This is because MIC can effectively detect non-linear associations like associations between decaying oscillation and the reference sin curve (Fig. 1). Although we compared the performance for only cosine wave, additional experiment with peak wave or complex wave is also important. ARS showed high performance following MICOP because de-trending at preprocessing seemed to cancel out the decay of time-series data. JTK was the tool with the third best detection power, although high performance was expected because it was based on Kendall’s tau, which is a measure of rank correlation, and it did not depend on amplitude. This indicates that MICOP has excellent performance for decaying oscillation, and suggests that an MIC-based approach that can detect non-linear associations is useful to detect decaying oscillation.

Moreover, we compared the MCC values for all methods on data containing gradual Gaussian noise to test the noise resistance (Fig. 3a). As a result, MICOP showed equal performance to JTK and ARS in the range of standard deviation of 0.125–0.500. This indicated that the performance of MICOP for noisy data is equal to that of the existing methods. This result suggests that the robustness to noise of MICOP is the same as that of well-known ARS and JTK, while the high performance of LS was limited to conditions with a low noise level. This numerical experiment revealed that the noise resistance of MICOP is the same as that of other widely used methods.

Clarifying the relationship between accuracy and sampling frequency in analyzing *omics* data, for which increasing the number of sampling points seems difficult, is important for determining the experimental design. As expected, with increase in the sampling frequency, the MCC values tended to increase (Figs. 2 and 3b). The fact that the ARS, JTK, and LS could characterize oscillation and non-oscillation in almost all cases when the sampling interval was 2 h or less is similar to the findings in original research studies of various methods and research comparing them [11, 28]. This suggested that a high sampling frequency improved accuracy; therefore, sampling frequency should be as high as experimental constraints allow.

We applied MICOP and existing methods for one-cycle of data (Fig. 4). As expected, accuracy decreased for all methods when one-cycle was used. However, MICOP and JTK showed high MCC values among methods under this condition. Also, MICOP seems to outperformed JTK under limited conditions which is low sampling frequency and high noise for one-cycle data (Fig. 4). Human *omics* data often have lower sampling frequencies, high noise levels, and only one-cycle. Our results suggest that MICOP and JTK have considerable potential for analyzing human *omics* datasets.

We reanalyzed the time-series proteomics data of C57BL/6 J and C57BL/6 to test the performance of MICOP and explore additional candidates of proteins with rhythmic change in their expression profile [8, 9]. These datasets include the mouse liver proteome data obtained by sampling every 3 h for 2 days, for which the analysis of the peptides was performed with a mass spectrometer. Approximately, 3000 protein types were detected in each study. Proteins that were detected in both MICOP and the original studies numbered 2 and 8 for C57BL/6 J and C57BL/6, respectively (Fig. 5). This actual application for proteomics data suggests that MICOP can obtain results in a manner approximately similar to the existing methods. Specifically, the MICOP results were consistent with those in the original articles regarding these commonly identified proteins. Furthermore, the proteins that were uniquely identified with MICOP were numbered 30 and 14 for C57BL/6 J and C57BL/6, respectively (Table 1, Table 2). These results strongly suggest that MICOP is a powerful tool to detect proteins with rhythmic changes in their expression levels from time-resolved proteomics data.

Although mass spectrometry-based approaches have been used for proteome-level studies of circadian rhythms, completely measuring mouse proteomes remains difficult. A comprehensive transcriptome analysis with parallel sequencers has revealed that ~ 15–20% of mouse liver mRNA significantly oscillates [29]. However, in these proteome studies of C57BL/6 and C57BL/6 J, significantly oscillating protein are rare (< 1% of detected total proteins; FDR < 0.05), a result inconsistent with those of mouse proteome studies. Multiple factors can explain this pattern. Typical clock protein known as principle oscillators such as CRY1, CRY2, PER2, REV-ERBα and CLOCK have comparatively low expression levels and are not detected in these studies [8, 9]. In addition, non-Gaussian experimental noise which is specific to MS measurement hampers the application of statistical test on proteins [30]. These problems may be improved by analyzing higher quality proteome datasets with modern technologies [31, 32]. Some core circadian proteins such as CRY1, CRY2, PER2, REV-ERVα and CLOCK could be detected in recently published proteome datasets [31, 32]. Thus, the development of proteome analysis technology may resolve discrepancies between results of transcriptome analysis and proteome analysis, and clarify connections within the circadian rhythm transcription and translation network.

We present a new list of proteins that oscillate by MICOP (Tables 1 and 2). The accuracy of these estimates is difficult to ascertain. Interestingly, when examining expression patterns of genes encoding these proteins, we estimated that the proteins were new oscillating molecules in MICOP. In addition, a large fraction of candidates was presumed to oscillate in a previous transcriptome analysis [29]. Two independent studies which measured both transcriptome and proteome of human samples revealed that only 30% of mRNA-protein correlation had statistically significant [33, 34]. This fact suggested that even if mRNA abundance is oscillating, protein abundance may not be always oscillating. However, about 90% of mRNA-protein correlation showed positive, hence rhythmic mRNA expression suggests the possibility of protein oscillation [34]. An overlap between re-analyzed proteomics data by MICOP and transcriptome analysis showed a consistent result.

MICOP accuracy tends to be low for data that do not perfectly fit a sine curve. The periodicity that MICOP can detect is subject to the shape of the reference curve, so changing the reference curve is necessary to detect asymmetric waveforms including saw tooth-like shapes like RAIN [30]. Furthermore, adjusting the false discovery rate is essential for accurate prediction, since MICOP repeats the hypothesis tests. In addition, verification with additional data such as periodic peak wave or overlapping sine wave is necessary in order to evaluate the accuracy of MICOP more precisely. Judgments of phase and cycle are possible in principle, but we did not perform them; therefore, this should be considered in future studies. Mutual information increased when sample size was small and correlation between two variables was null, even when the variables were random [35]. We solved this issue in MICOP by determining the *P*-value with the Monte Carlo method. When the time points (sample size) are small, the criterion for calculating the *P*-value increases, and when the time points are large, the criterion for calculating the *P*-value decreases (Additional file 3). In this paper, we presented MICOP, which is an MIC-based algorithm, for predicting periodic patterns in large-scale time-resolved protein expression profiles. The performance test using artificially generated simulation data revealed that the performance of MICOP for decaying data was superior to that of the existing widely used methods. Additionally, we indicated that MICOP is compatible with noisy data obtained with a low sampling frequency. Furthermore, the performance test using actual mouse proteomics data suggested that MICOP may be able to provide novel findings from proteomics data. Specifically, it can reveal novel findings from time-series data and may contribute to biologically significant results. This study suggests that MICOP is an ideal approach for detecting and characterizing oscillations in time-resolved *omics* data sets.

## Conclusion

In this paper, we presented MICOP, which is an MIC-based algorithm, for predicting periodic patterns in large-scale time-resolved protein expression profiles. The performance test using artificially generated simulation data revealed that the performance of MICOP for decaying data was superior to that of the existing widely used methods. Additionally, we indicated that MICOP is compatible with noisy data obtained with a low sampling frequency. Furthermore, the performance test using actual mouse proteomics data suggested that MICOP may be able to provide novel findings from proteomics data. Specifically, it can reveal novel findings from time-series data and may contribute to biologically significant results. This study suggests that MICOP is an ideal approach for detecting and characterizing oscillations in time-resolved *omics* data sets.

## Additional files

Additional file 1: Wide range comparison of MCC values of MICOP, ARS, JTK, and LS for decaying data. Sampling interval and noise level were gradually adjusted. The bar indicates MCC values (1 indicates a perfect prediction, 0 indicates a random prediction, and − 1 indicates a prediction in complete disagreement). (PDF 75 kb)

Additional file 2: Wide-range comparison of MCC values of MICOP, ARS, JTK, and LS for non-decaying data. Sampling interval and noise level were gradually adjusted. The bar indicates MCC values (1 indicates a perfect prediction, 0 indicates a random prediction, and − 1 indicates a prediction in complete disagreement). (PDF 75 kb)

Additional file 3: Monte-Carlo simulation to calculate *P*-values. MIC values were calculated between random numbers. The x-axis indicates sample number (N time points) and the y-axis indicates MIC. The error bar indicates the standard deviation (*N* = 1000). The red color represents random values and the blue color represents the significance threshold (5%). (PDF 68 kb)

## Abbreviations

ARS: Autoregressive spectral estimation
FN: False Negative
FP: False positive
JTK: Jonckheere–Terpstra (JT) test and obtained the strength of correlation by Kendal’s tau test
LS: Lomb-Scargle
MCC: Mathews correlation coefficient
MIC: Maximal information coefficient
MICOP: Maximal information coefficient-based oscillation prediction
MINE: Maximal information-based nonparametric estimation
TN: True negative
TP: True positive
