# Supplementary figures and images for: MICOP: Maximal information coefficient-based oscillation prediction to detect biological rhythms in proteomics data
Source: BMC Bioinformatics. 2018 Jun 28;19:249. doi: 10.1186/s12859-018-2257-4 (PMC6025708; doi:10.1186/s12859-018-2257-4)

Sampling interval (hours)

MCC

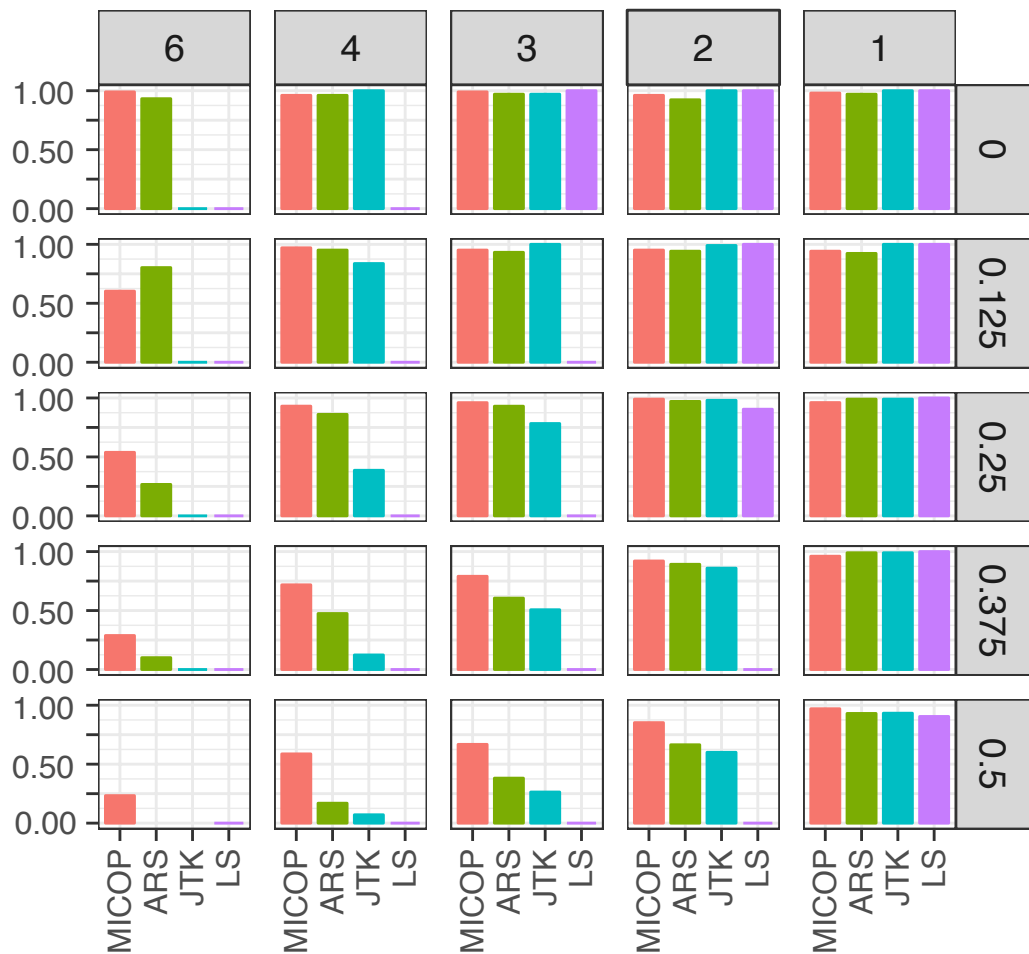

Noise level

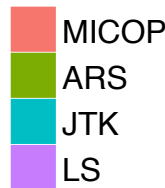

Supplement: Supplementary file 1 — Wide range comparison of MCC values of MICOP, ARS, JTK, and LS for decaying data. Sampling interval and noise level were gradually adjusted. The bar indicates MCC values (1 indicates a perfect prediction, 0 indicates a random prediction, and − 1 indicates a prediction in complete disagreement). (PDF 75 kb) [file 12859_2018_2257_MOESM1_ESM.pdf]

Sampling interval (hours)

MCC

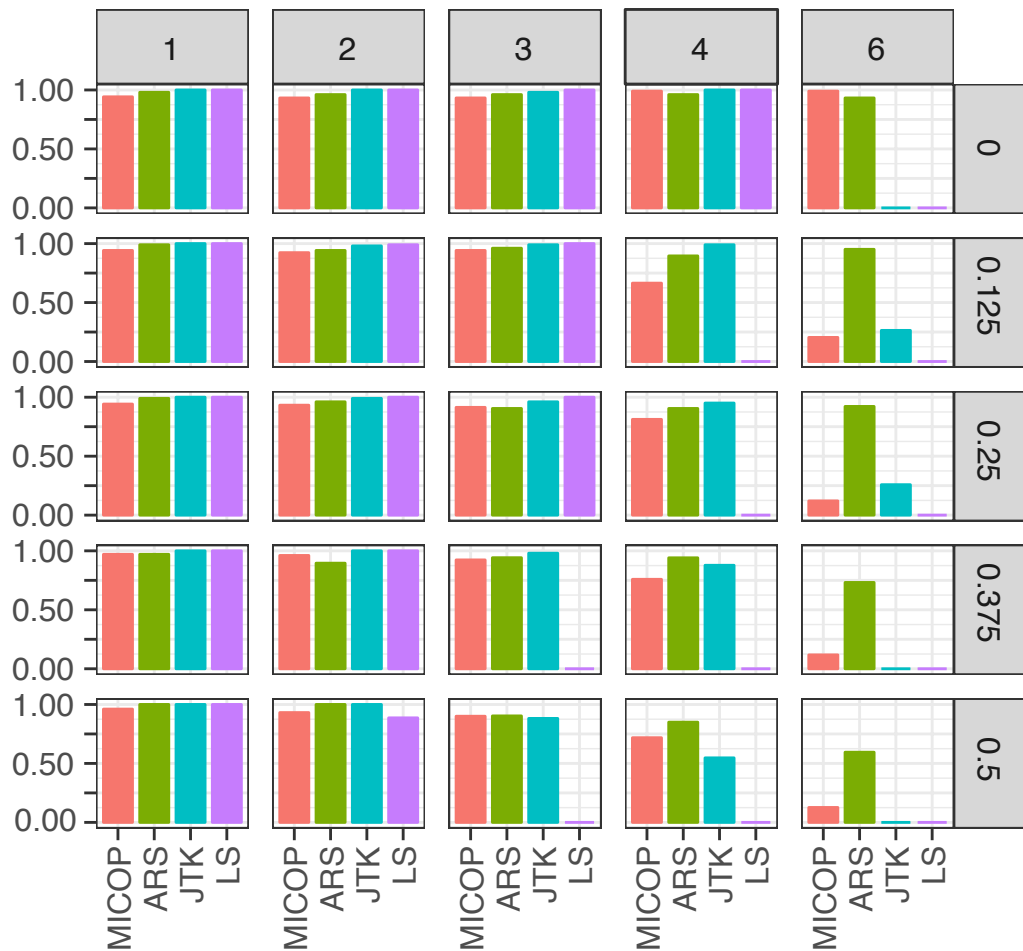

Noise level

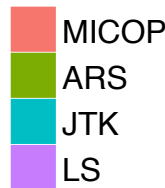

Supplement: Supplementary file 2 — Wide-range comparison of MCC values of MICOP, ARS, JTK, and LS for non-decaying data. Sampling interval and noise level were gradually adjusted. The bar indicates MCC values (1 indicates a perfect prediction, 0 indicates a random prediction, and − 1 indicates a prediction in complete disagreement). (PDF 75 kb) [file 12859_2018_2257_MOESM2_ESM.pdf]

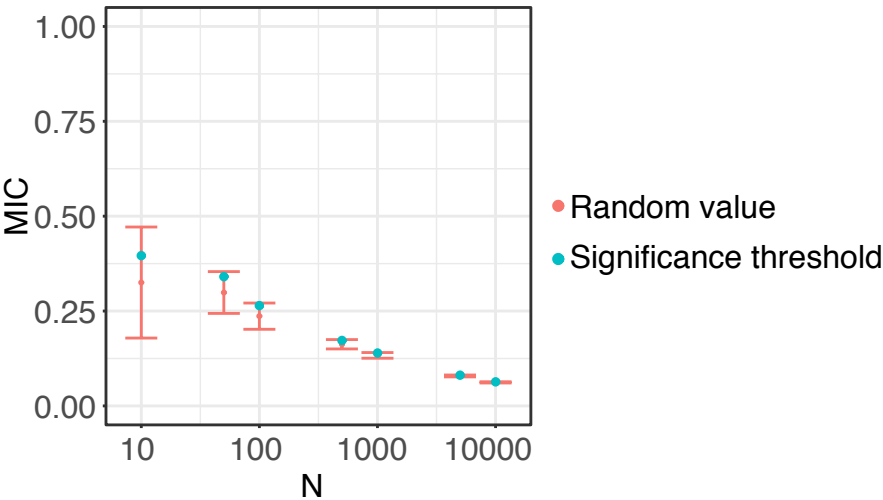

Supplement: Supplementary file 3 — Monte-Carlo simulation to calculate P-values. MIC values were calculated between random numbers. The x-axis indicates sample number (N time points) and the y-axis indicates MIC. The error bar indicates the standard deviation (N = 1000). The red color represents random values and the blue color represents the significance threshold (5%). (PDF 68 kb) [file 12859_2018_2257_MOESM3_ESM.pdf]
